# Indium‐Iron Oxide Nanosized Solid Solutions as Photocatalysts for the Degradation of Pollutants under Visible Radiation

**DOI:** 10.1002/cssc.202401180

**Published:** 2024-11-07

**Authors:** Damiano Cani, Timo Cuyvers, Paolo P. Pescarmona

**Affiliations:** ^1^ Centre for Surface Chemistry and Catalysis University of Leuven (KU Leuven) Kasteelpark Arenberg 23 3001 Heverlee Belgium; ^2^ Chemical Engineering Group Engineering and Technology Institute Groningen (ENTEG) University of Groningen Nijenborgh 4 9747 AG Groningen The Netherlands

**Keywords:** Photocatalysis, solid solutions, indium-iron oxide, visible light, degradation of pollutants, water purification

## Abstract

A series of solid solutions of indium and iron oxides with different In/Fe ratios (In_x_Fe_y_O_3_, with x + y = 2) were synthesized in the form of nanoparticles (diameter of ca. 30–40 nm) with the purpose of generating enhanced photocatalysts with an intermediate band gap compared to those of the monometallic oxides, In_2_O_3_ and Fe_2_O_3_. The materials were prepared by co‐precipitation from an aqueous solution of iron and indium nitrates and extensively characterized with a combination of techniques. XRD analysis proved the formation of the desired In_x_Fe_y_O_3_ solid solutions for Fe content in the range 5–25 mol%. UV‐Vis absorption analysis showed that the substitution of In with Fe in the crystalline structure led to the anticipated gradual decrease of the band gap values compared to In_2_O_3_. The obtained semiconductors were tested as photocatalysts for the degradation of model organic pollutants (phenol and methylene blue) in water. Among the In_x_Fe_y_O_3_ solid solutions, In_1.7_Fe_0.3_O_3_ displayed the highest photocatalytic activity in the degradation of the selected probe molecules under UV and visible radiation. Remarkably, In_1.7_Fe_0.3_O_3_ showed a significantly enhanced activity under visible light compared to monometallic indium oxide and iron oxide, and to the benchmark TiO_2_ P25. This demonstrates that our strategy consisting in engineering the band gap by tuning the composition of In_x_Fe_y_O_3_ solid solutions was successful in improving the photocatalytic performance under visible light. Additionally, In_1.7_Fe_0.3_O_3_ fully retained its photocatalytic activity upon reuse in four consecutive cycles.

## Graphical abstract

Indium‐iron oxide nanosized solid solutions are a novel class of photocatalysts that efficiently utilize visible light to promote the degradation of organic pollutants for water purification. These photocatalysts display a significantly enhanced activity compared to monometallic indium oxide and iron oxide, and can be reused without loss of activity.

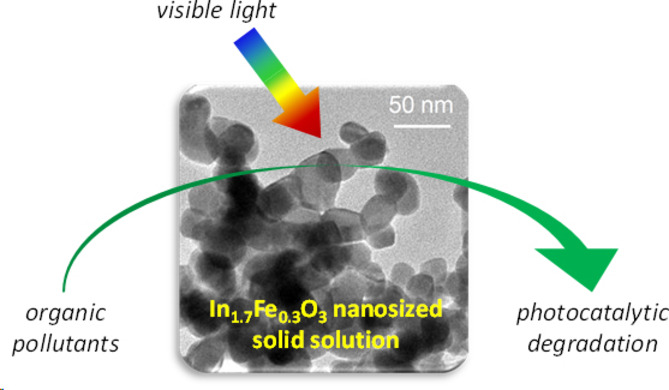

## Introduction

The use of metal oxides as photocatalysts has been extensively investigated since the work of Fujishima and Honda in 1972, in which water splitting was achieved over a titanium dioxide photoelectrode.^[1]^ Since then, several applications for photocatalytic systems have been developed, such as the reduction of CO_2_ to hydrocarbons and the degradation of organic pollutants for water purification.[^2^, ^3^, ^4^, ^5^, ^6^, ^7^] Focusing on the latter application, different techniques such as filtration, adsorption, sedimentation or microbiological degradation have been reported for the purification of water streams. However, photocatalysis has some striking advantages compared to the classically employed techniques, as it can deal with a variety of compounds (including antibiotics that cannot be degraded in microbiological processes) and ideally it can convert the pollutant to CO_2_ and water obtaining a complete degradation of the organic molecule (which is only moved to another medium in the case of filtration, adsorption or sedimentation). Additionally, when utilizing photocatalysts that are active upon absorption of solar radiation, in principle no energy input is required to achieve the degradation.

For practical application in water purification, heterogeneous photocatalysts are preferable over their homogeneous counterparts as they do not require separation from the purified water. Heterogeneous photocatalysts are based on semiconductors, and operate by exciting electrons from the valence band to the conduction band upon absorption of radiation having higher energy than the band gap energy. The obtained loosely bound, excited electrons and the holes they leave behind can take part in redox reactions and thus represent the photocatalytically active species.^[8]^ In the photocatalytic degradation of pollutants in water, the electron‐hole couples typically react with O_2_ and H_2_O to form radical species such as the superoxide O_2_^−⋅^ and the hydroxyl radical OH^⋅^, which in turn react with the pollutants located at the surface of the photocatalyst or in its vicinity, leading to their oxidative degradation.[^9^, ^10^]

TiO_2_ is the most studied heterogeneous photocatalyst due a favorable combination of properties: it is inexpensive, stable, non‐toxic and shows a good activity under UV irradiation.[^11^, ^12^, ^13^, ^14^, ^15^, ^16^, ^17^] The main disadvantage in the use of TiO_2_ as photocatalyst is connected to its band gap of 3.0–3.2 eV, which allows excitation with UV radiation but not with visible light (1.65 eV < hν < 3.00 eV), making it inefficient for operating with solar radiation (composed largely by visible light and only for around 5 % by UV radiation). Many efforts have been undertaken to develop visible‐light‐active TiO_2_ photocatalysts, by doping the oxide with metallic or non‐metallic atoms and by photosensitization with organic compounds.[^18^, ^19^, ^20^, ^21^, ^22^, ^23^] However, issues connected to poor stability (when a dye is used as sensitizing agent) and relatively low activity due to increased recombination (when doping agents are inserted in the TiO_2_ matrix to promote visible light absorption) prompted the quest for other photocatalysts that can operate with visible light radiation.[^24^, ^25^, ^26^]

Among metal oxide photocatalysts, much attention has been given to iron oxide (Fe_2_O_3_),[^27^, ^28^, ^29^, ^30^] which is based on earth‐abundant elements, is inexpensive and has a band gap energy (1.9–2.2 eV) that permits absorption of visible light.[^31^, ^32^] However, the photocatalytic performance of Fe_2_O_3_ is limited by a number of intrinsic issues. First, the defects commonly found in its crystal structure act as recombination centers for the electron‐hole couples, preventing them from leading to a redox reaction at the surface of the material.^[33]^ Second, the charge carriers move through the material by Fe^3+^/Fe^2+^‐localized valence alteration, which leads to a low mobility of the photogenerated species.^[34]^ Finally, the position of the conduction band of Fe_2_O_3_ is at too low energy (E_cb_ = +0.2 eV)^[35]^ to efficiently promote the reduction of O_2_ to O_2_^−⋅^, which is an important reactive oxygen species involved in the photocatalytic degradation of pollutants.^[36]^ These issues have been partially overcome with the synthesis of morphologically‐engineered iron oxide materials, but TiO_2_ is reported to still outperform iron–based photocatalysts especially under UV irradiation.[^37^, ^38^] Indium oxide (In_2_O_3_) is a less studied material for photocatalytic applications; it is a semiconductor with band gap energy in the range 2.9–3.5 eV (E_cb_ = −0.5 eV), whose band structure has been extensively discussed, but without reaching a consensus on the nature of the electronic transition at the root of the optical absorption.[^39^, ^40^, ^41^] In any case, the band gap width implies that radiation in the UV range is needed for the application of In_2_O_3_ as photocatalyst. The reported photocatalytic applications of In_2_O_3_ are focused on the degradation of organic pollutants.[^42^, ^43^] Morphological control on the material to increase the surface area and favor the adsorption of the pollutants is the most used approach to promote its photocatalytic activity.^[44]^ On the other hand, modifications of In_2_O_3_ to produce a visible‐light active photocatalyst are poorly investigated, with the main approaches being the formation of heterojunctions or doping.[^45^, ^46^, ^47^, ^48^] To the best of our knowledge, the extension of the absorption of In_2_O_3_ into the visible light, without the use of sensitizers, has not been reported so far.

Here, we report a novel strategy for engineering the band gap of In_2_O_3_ by forming solid solutions with Fe_2_O_3_. This novel class of materials combines enhanced photocatalytic activity under visible light with the high stability that is characteristic of solid solutions. While the use of mixed oxides with spinel or perovskite structures for photocatalytic applications with visible light has been studied extensively (e.g. BiVO_4_ and Bi_2_WO_6_),[^49^, ^50^, ^51^, ^52^, ^53^, ^54^] the strategy we adopted in this work, consisting in developing solid solutions to produce photocatalysts with activity in the visible region, is much less explored[^55^, ^56^] with no reports of the In−Fe oxide solid solutions we investigated here. The formation of a solid solution between two oxides in the whole range of compositions generally requires satisfying the Hume‐Rothery rules: (1) the solute and solvent ionic radii should not differ for more than 15 %; (2) the crystal structure of the two oxides should be the same; (3) the metal cations should have the same valence numbers; and (4) similar electronegativity. In the case of indium oxide and iron oxide, both the cations have +3 oxidation state, similar electronegativities (1.83 for iron and 1.78 for indium) and the ionic radii (0.645 Å for Fe^3+^ and 0.80 Å for In^3+^) differ for 19 %.[^47^, ^57^] Indium oxide crystallizes in a body centered cubic crystal structure,^[58]^ while the thermodynamically stable iron oxide phase is rhombohedral (α‐Fe_2_O_3_), although this compound can also be prepared as a body‐centered cubic metastable polymorph.[^59^, ^60^] The difference in the ionic radii above 15 % and the different thermodynamically stable crystal structure anticipate that the formation of solid solutions of the two materials in the whole range of compositions is unlikely to be feasible. Nonetheless, the other favorable features suggest the possibility of achieving In_x_Fe_y_O_3_ solid solutions for In‐rich materials (x > y, with x + y = 2) that are expected to be able to host a small fraction of iron into the cubic indium oxide structure without modification of the crystal phase.

Solid solutions display intermediate properties between those of the pure materials.^[61]^ Therefore, we anticipated that preparing In_x_Fe_y_O_3_ solid solutions would allow tuning the band gap to intermediate values compared to those of pure In_2_O_3_ and Fe_2_O_3_. In this way, we aimed at increasing the visible‐light absorption compared to In_2_O_3_ and to improve the band positions compared to Fe_2_O_3_. Additionally, the controlled co‐precipitation method that we employed to prepare the In_x_Fe_y_O_3_ solid solutions allowed obtaining these materials in the form of nanoparticles. This combination of desirable features led to photocatalysts with enhanced performance in the degradation of probe pollutants (phenol and methylene blue) under visible radiation compared to In_2_O_3_ and Fe_2_O_3_ and to the benchmark TiO_2_ P25 photocatalyst.

## Results and Discussion

### Synthesis and Characterization of the In_x_Fe_y_O_3_ Solid Solutions

**Figure 1:**
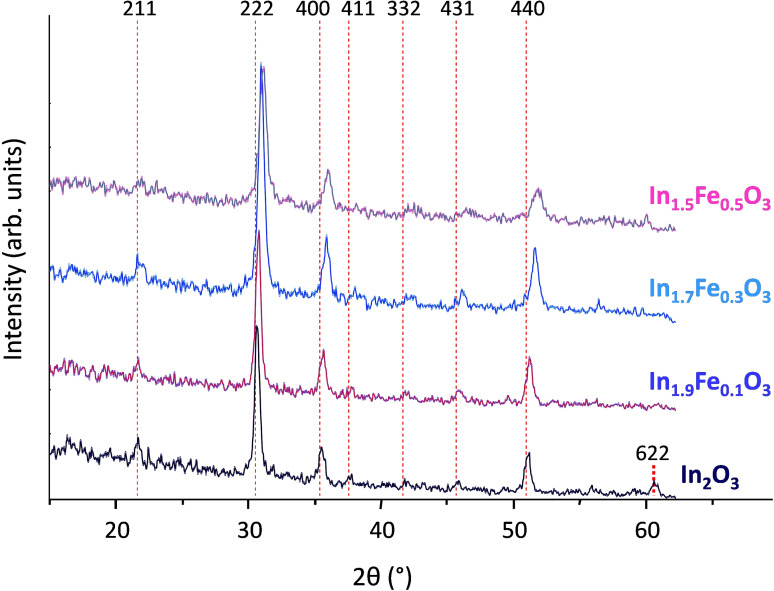
Diffractograms of the synthesized In_x_Fe_y_O_3_ materials and of In_2_O_3_. All the peaks can be indexed with the structure of cubic In_2_O_3_. The dotted lines help in the visualization of the peaks shift towards higher 2θ (°) when the iron fraction in the solid solution is increased.

**Figure 2:**
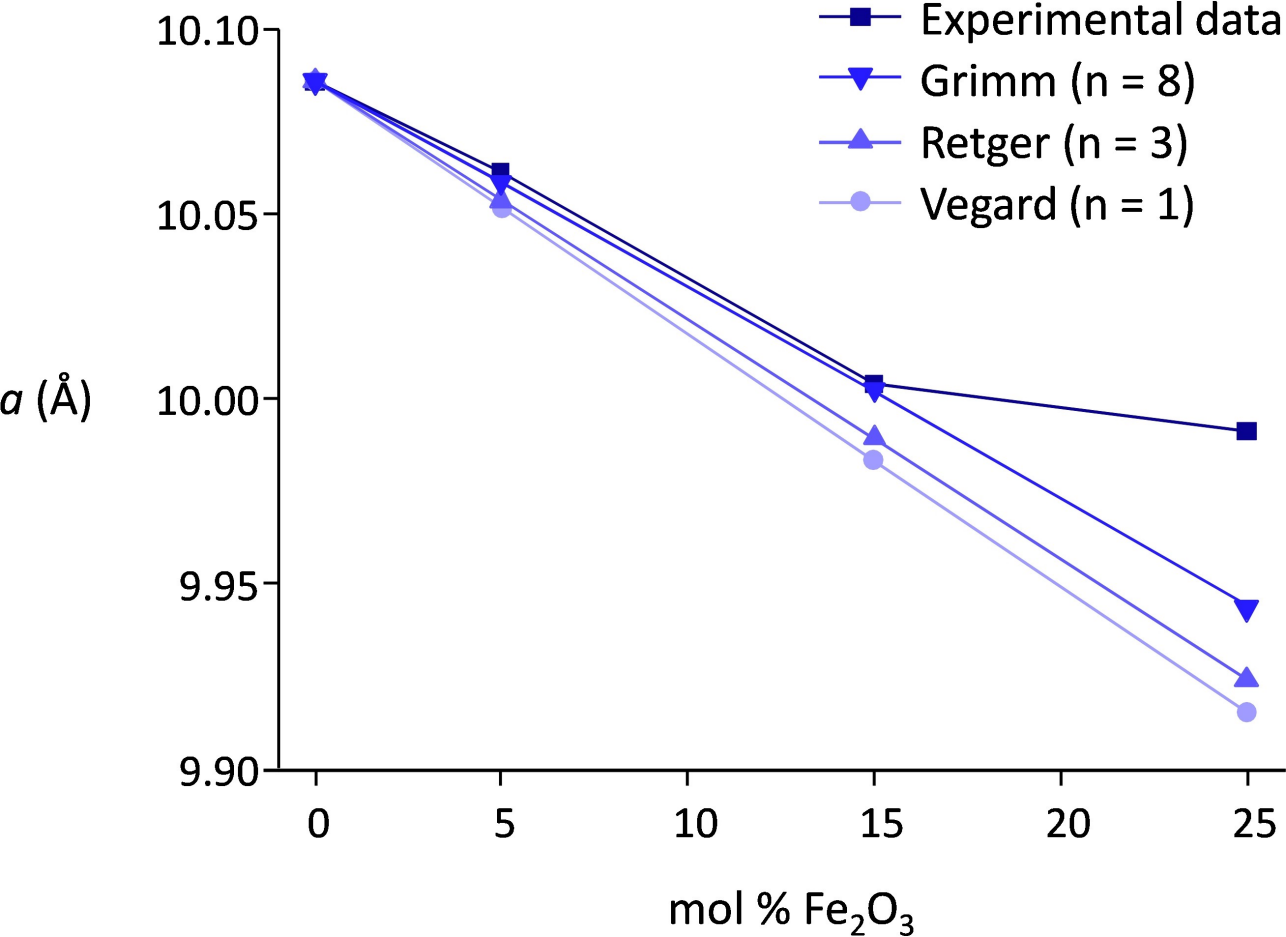
Lattice parameter *a* (Å) evaluated from the (222) peak in the In_x_Fe_y_O_3_ series (with y = 0, 0.1, 0.3, 0.5). The experimental values are plotted together with the expected values based on three different theoretical equations (Vegard, Retger, Grimm). See Supporting Information for the equation used for each model. The same trend observed in this figure was found when other diffraction peaks were considered (see Figures S2 and S3).

A series of nanosized solid solutions of iron and indium oxide in the In‐rich region (In_x_Fe_y_O_3_, with x + y = 2 and x = 1.9, 1.7, 1.5) was synthesized by a controlled co‐precipitation method involving the gradual addition of ammonium carbonate to an aqueous solution of indium and iron nitrates. XRD analysis was carried out to determine whether the desired solid solutions were successfully prepared.[^62^, ^63^] The diffractogram of indium oxide prepared with the same method used to synthesize the In_x_Fe_y_O_3_ solid solutions (Figure 1), shows peaks corresponding to the body‐centered cubic polymorph of In_2_O_3_ (file 00–006‐0416 PDF‐2008). Indium oxide can also crystallize in a metastable rhombohedral phase that transforms into the thermodynamically stable cubic phase upon thermal treatment.[^40^, ^64^] The indium oxide prepared in this work was subjected to calcination at 700 °C and, accordingly, its diffractogram presents only signals from the cubic phase. Remarkably, the mixed oxides prepared with up to 25 mol% of Fe presented the same body‐centered cubic crystal structure and no separate crystalline iron oxide phase was detected. Yet, an important difference was observed: the diffraction peaks of the In_x_Fe_y_O_3_ samples displayed a gradual shift towards higher 2θ° values upon increasing Fe content in the material (Figure 1). This is a clear indication that Fe was incorporated in the body centered cubic lattice characteristic of In_2_O_3_, with the increase in the 2θ values being a consequence of a slight shrinking of the lattice (according to Bragg′s law, n*λ* = 2*d* sinθ) caused the smaller ionic radius of Fe^3+^ compared to In^3+^. This unequivocally proves that the desired solid solutions were successfully synthesized. The miscibility limit for the formation of In_x_Fe_y_O_3_ solid solutions was found to be between 25 and 40 mol% of iron oxide in the structure of indium oxide, as indicated by the presence of diffraction peaks of rhombohedral α‐Fe_2_O_3_ together with those of cubic In_2_O_3_ in the material prepared with a ratio 6 : 4 between In and Fe (Figure S1). The variation of the lattice parameter for the cubic In_x_Fe_y_O_3_ solid solutions as a function of the molar content of Fe was evaluated based on different models (Vegard, Retger or Grimm, see SI for more information).[^65^, ^66^] The best fit of our experimental results was found with the model proposed by Grimm (Figure 2), with an excellent matching between model and experimental data up to an iron molar fraction of 15 % (y = 0.3). Only In_1.5_Fe_0.5_O_3_ deviates substantially from the model, probably because not all the iron is incorporated in the indium oxide structure and instead is present as amorphous (XRD silent) iron oxide domains.

**Figure 3:**
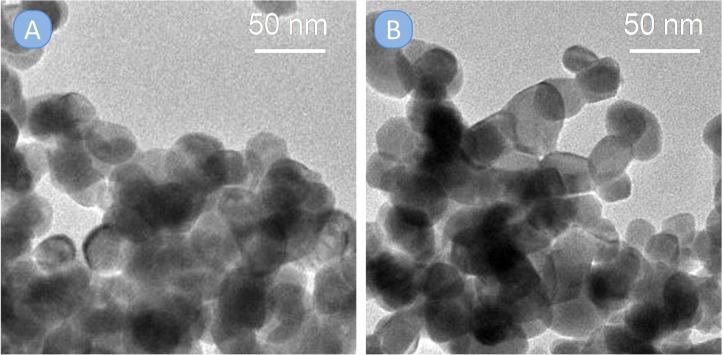
TEM images of selected In_x_Fe_y_O_3_ oxides: In_1.9_Fe_0.1_O_3_ (A) and In_1.7_Fe_0.3_O_3_ (B).

The controlled co‐precipitation method used to prepare the In_x_Fe_y_O_3_ solid solutions led to the formation of ellipsoidal nanoparticles with a rather uniform size of ca. 30–40 nm, as shown by TEM (Figure 3). No porosity was observed in the TEM images, except for the interparticle voids. The In and Fe contents measured by EDX were in good agreement with the theoretical values, showing 83 mol% of In_2_O_3_ in In_1.7_Fe_0.3_O_3_ and 74 mol% in the case of In_1.5_Fe_0.5_O_3_. The EDX spectrum of In_1.9_Fe_0.1_O_3_ was not informative about the presence of Fe, because of its small fraction in this material.

**Figure 4:**
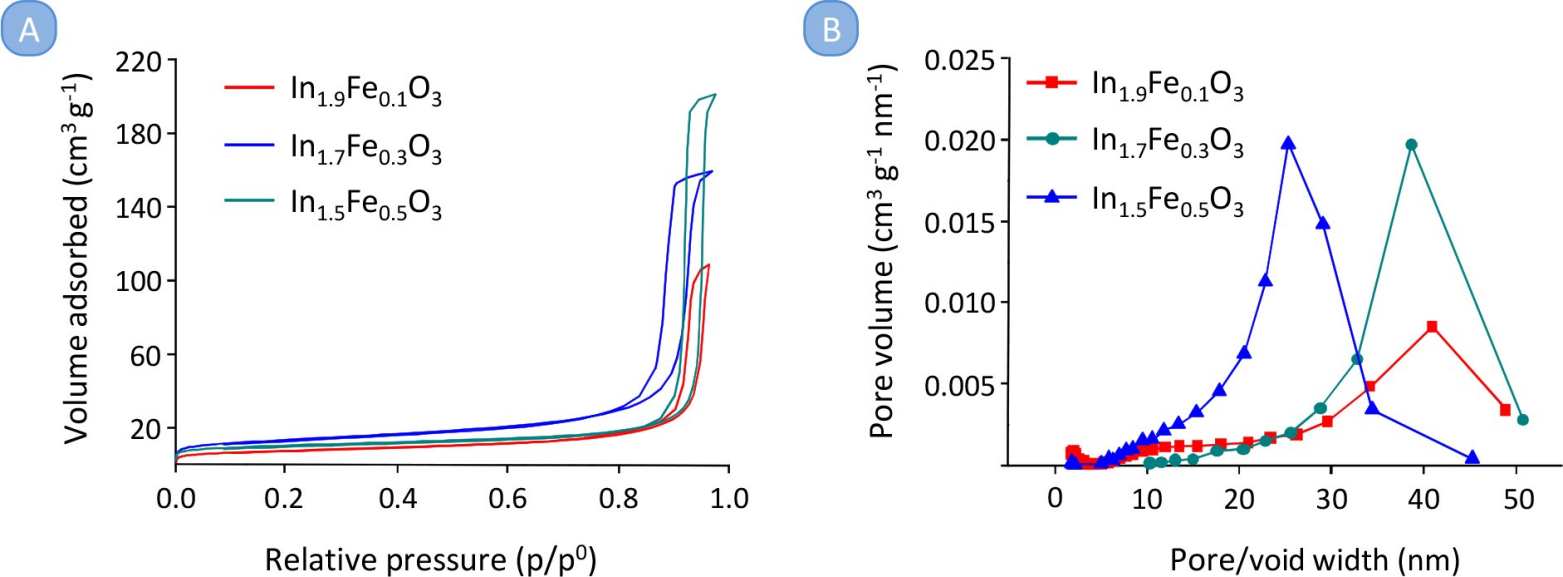
Nitrogen physisorption isotherms (left) and pore (void) size distribution (right) for the In_x_Fe_y_O_3_ solid solutions.

The textural properties of the In_x_Fe_y_O_3_ solid solutions were determined by N_2_ physisorption (Figure 4). All isotherms are of type IV with H1 hysteresis loop. The pore size distributions are broad and span the region between 10 and 50 nm (mesopore region), but rather than stemming from structural porosity are originating from the interparticle voids evidenced by TEM.^[67]^ The BET specific surface areas for the solid solutions are 25 m^2^/g for In_1.9_Fe_0.1_O_3_, 45 m^2^/g for In_1.7_Fe_0.3_O_3_ and 35 m^2^/g for In_1.5_Fe_0.5_O_3_, which are in the same order of magnitude as the widely used benchmark photocatalyst TiO_2_ P25, which has similar particle size and shows a surface area of around 55 m^2^/g.[^68^, ^69^]

**Figure 5:**
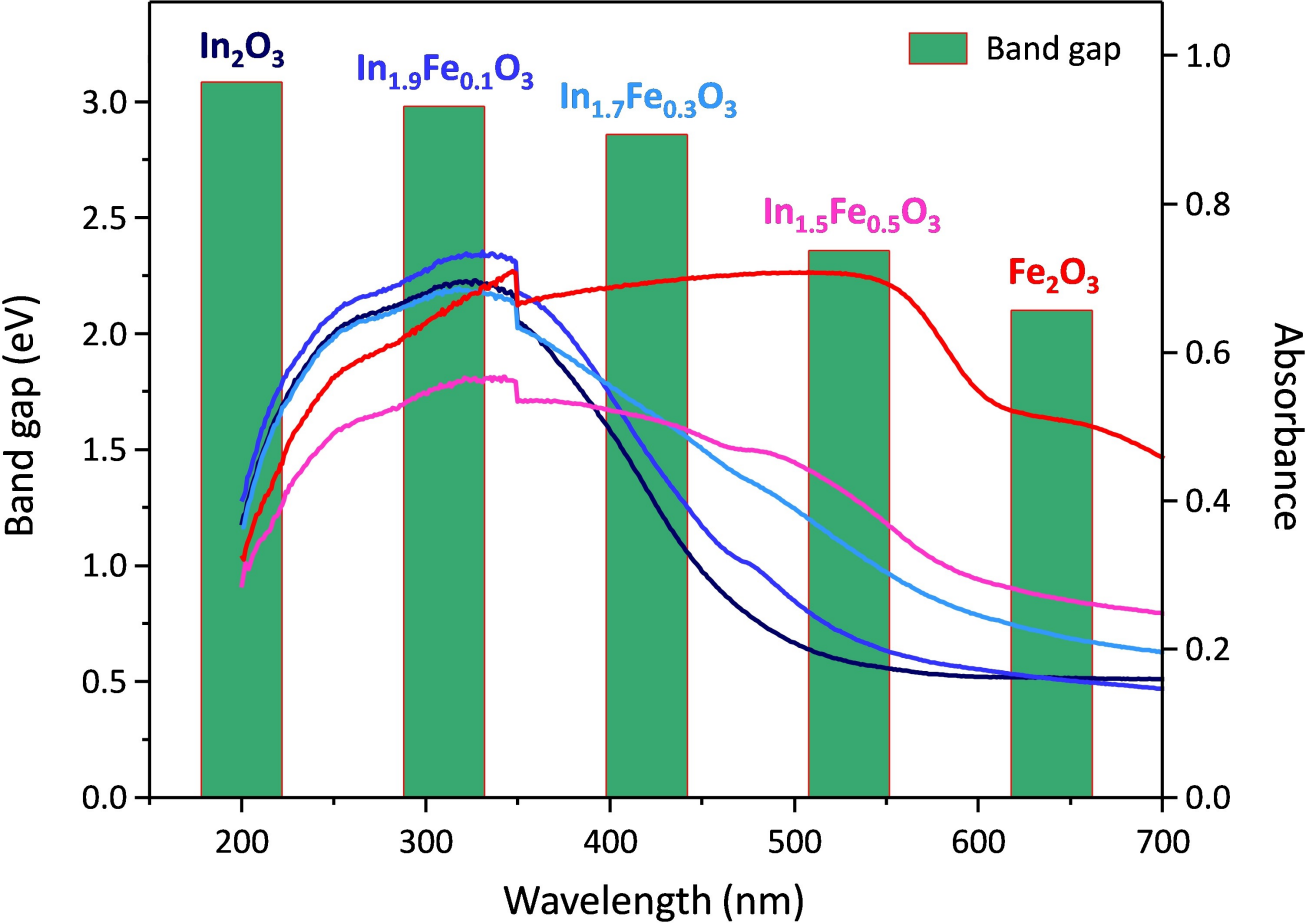
Solid state UV‐Vis absorption spectra of the In_x_Fe_y_O_3_ solid solutions, In_2_O_3_ and Fe_2_O_3_ (lines); and band gaps of the same materials evaluated from the absorption profiles using the Tauc plot method for a direct transition (green bars). The steep increase in the absorption at around 350 nm is an artifact due to the switch in the reflection mirror in the instrument.

The incorporation of Fe(III) in the In_2_O_3_ lattice is expected to influence the electronic band structure of the obtained solid solutions. Therefore, the optical properties of the In_x_Fe_y_O_3_ solid solutions were investigated by solid state UV‐Vis absorption spectroscopy in diffusive reflectance mode (Figure 5). Notably, the absorption edge in the spectra of the solid solutions gradually extends towards the visible region compared to that of In_2_O_3_, with the shift increasing with the amount of iron in the material. This indicates a gradual modification of the band structure in the formed solid solutions brought about by the presence of iron. Yet, the absorption edge of pure Fe_2_O_3_ (reported as reference in Figure 5) is at higher wavelengths.^[70]^ Consistently with these observations, the band gap energies of the In_x_Fe_y_O_3_ materials are intermediate between those found for Fe_2_O_3_ and In_2_O_3_ (2.06 and 3.09 eV, respectively, in agreement with the values reported in literature [26, 36]) and gradually increase with increasing iron content in the material. The band gaps of all In_x_Fe_y_O_3_ solid solutions fall in the visible region, which is an attractive feature for the application of these materials as photocatalysts with activity under solar radiation. Against this backdrop, the synthesized materials were tested as photocatalysts for the degradation of two probe pollutants (phenol and methylene blue) in water solution under UV and visible radiation.

### Photocatalytic Activity of the In_x_Fe_y_O_3_ Solid Solutions

**Figure 6:**
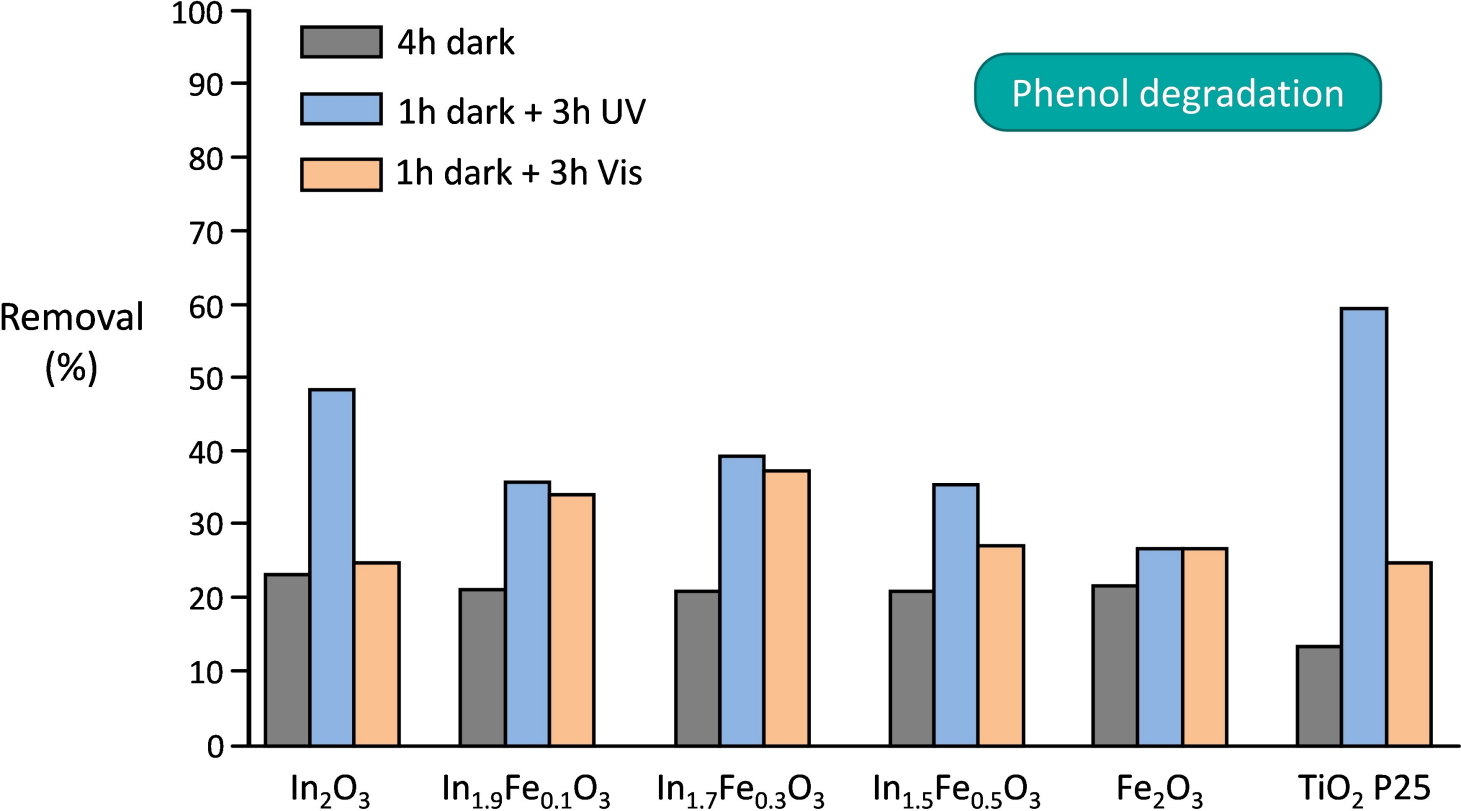
Photocatalytic degradation of phenol over In_x_Fe_y_O_3_, In_2_O_3_, Fe_2_O_3_ and the benchmark TiO_2_ P25. Conditions: 50 ppm phenol (5 ml), 15 mg of photocatalyst, 1 h adsorption in the dark followed by 3 h of irradiation, or 4 h adsorption in the dark. The tests were performed at 35 °C in aerobic conditions.

**Figure 7:**
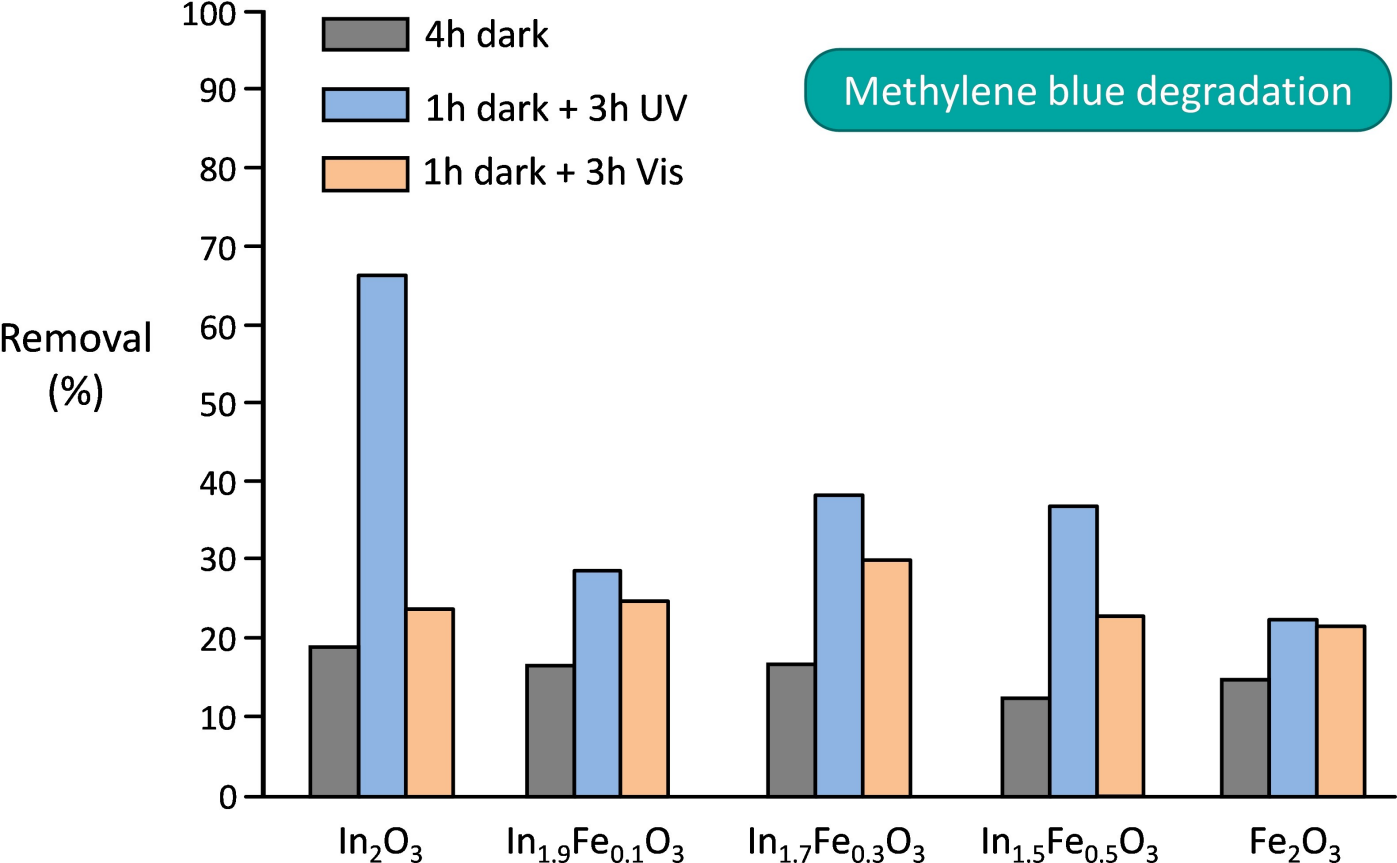
Photocatalytic degradation of methylene blue over In_x_Fe_y_O_3_, In_2_O_3_, Fe_2_O_3_ and the benchmark TiO_2_ P25. Conditions: 10 ppm methylene blue (5 ml), 15 mg of photocatalyst, 1 h adsorption in the dark followed by 3 h of irradiation, or 4 h adsorption in the dark. The tests were performed at 35 °C in aerobic conditions.

In the investigation of the performance of the In_x_Fe_y_O_3_ solid solutions as heterogeneous photocatalysts for the degradation of pollutants in aqueous solutions, phenol and methylene blue were chosen as probe compounds for the tests under either UV or visible radiation. The degradation of methylene blue or other industrial dyes is commonly used to assess the degradation activity of photocatalysts under visible light, although this practice has been criticized as the ability of dyes to absorb light in the visible region leading to sensitization effect can cause an overestimate of the activity of the photocatalysts.^[71]^ Based on these considerations, we chose to complement the use of the bulky, ionic methylene blue dye with that of phenol as a second probe pollutant, as the latter is a non‐ionic, smaller compound that is transparent to visible light. The photocatalytic activity of the In_x_Fe_y_O_3_ materials was compared to that of pure indium oxide and iron oxide, to highlight the differences in performance brought about by the formation of the solid solutions, and to that of TiO_2_ P25, which is often used as benchmark in photocatalytic tests.^[72]^ Since the adsorption of the probe compound on the surface of the photocatalyst is generally considered the first step in the degradation process (though the degradation may also proceed in the region adjacent to the surface by diffusion of the photocatalytically‐generated radicals), we started our study by quantifying the amount of probe compound adsorbed on the photocatalysts in the absence of radiation (4 h of stirring in the dark, Figures 6 and 7). Phenol is more efficiently adsorbed than methylene blue in all the dark tests, with no major differences in adsorption capacity among the In_x_Fe_y_O_3_ solid solutions. When the photocatalytic tests were performed under UV irradiation (after 1 h in the dark), indium oxide showed the best performance among the series of materials synthesized in this work, both in the degradation of phenol and methylene blue (Figures 6 and 7), but was still inferior compared to the benchmark TiO_2_ P25. The higher activity of In_2_O_3_ compared to the In_x_Fe_y_O_3_ solid solutions can be reasonably attributed to its larger band gap (3.09 eV, see Figure 5), which implies that more energetic electron‐hole couples are produced upon UV irradiation. Similarly, the higher activity of the In_x_Fe_y_O_3_ photocatalysts compared to α‐Fe_2_O_3_ is ascribed to their larger band gap, though an additional contribution could originate from the fact that these solid solutions are structurally related to In_2_O_3_ and might thus not suffer from (some of) the intrinsic limitations of α‐Fe_2_O_3_ (*vide supra*).[^33^, ^34^] Among the In_x_Fe_y_O_3_ solid solutions, In_1.7_Fe_0.3_O_3_ displayed a slightly higher photocatalytic activity under UV radiation than the other two materials, both in the degradation of phenol and methylene blue (Figures 6 and 7). The fact that In_1.7_Fe_0.3_O_3_ is slightly more active than In_1.9_Fe_0.1_O_3_ despite the slightly larger band gap of the latter can be ascribed to its larger specific surface area (*vide supra*).

The purpose of this work was to develop a new class of photocatalysts with enhanced activity compared to both Fe_2_O_3_ and In_2_O_3_ with visible radiation. This is highly desirable, because it would allow using the widely available solar radiation as energy source for promoting the degradation reactions leading to water purification. Our strategy involving the preparation of In_x_Fe_y_O_3_ solid solutions proved successful for achieving this target, as demonstrated by the photocatalytic tests under visible light irradiation (Figures 6 and 7). The In_x_Fe_y_O_3_ solid solutions, and more clearly In_1.7_Fe_0.3_O_3_ and In_1.9_Fe_0.1_O_3_, were found to be more active in the degradation of both phenol and methylene blue compared to the pure oxides, α‐Fe_2_O_3_ and In_2_O_3_, and to TiO_2_ P25. Their higher activity under visible light compared to In_2_O_3_ and TiO_2_ P25 is a direct consequence of the decrease in the band gap caused by the incorporation of Fe in the In_2_O_3_‐like cubic structure of these solid solutions (Figure 5), which implies that these materials display enhanced absorption of radiation in the visible range and thus show increased photocatalytic activity under these conditions. This is further highlighted by the almost identical photocatalytic activity shown by In_1.7_Fe_0.3_O_3_ and In_1.9_Fe_0.1_O_3_ under visible light compared to their performance under UV irradiation, while In_2_O_3_ and TiO_2_ P25 are much less active under visible light, as expected considering that their large band gap allows excitation mainly with UV radiation.^[73]^ On the other hand, α‐Fe_2_O_3_ has the smallest band gap value (2.06 eV) among the tested materials, which endows it with more efficient absorption of radiation in the visible range. Consequently, the photocatalytic activity of iron oxide was nearly the same in the tests under visible or UV irradiation (Figures 6 and 7). The higher photocatalytic activity of the In_x_Fe_y_O_3_ solid solutions, and particularly of In_1.7_Fe_0.3_O_3_, compared to α‐Fe_2_O_3_ despite the ability of the latter to absorb radiation in a larger range in the visible region, is attributed to a more suitable position of the conduction band of the In_x_Fe_y_O_3_ materials (which are structurally related to In_2_O_3_ ^[74]^), though other factors as a lower concentration of defects acting as recombination centers for the electron‐hole couples might also have contributed.^[35]^ Within this line of reasoning, it is expected that for the most active solid solutions, In_1.7_Fe_0.3_O_3_ and In_1.9_Fe_0.1_O_3_, the photocatalytic degradation of the probe pollutants involves the same reactive oxygen species generally reported for In_2_O_3_, i.e. the superoxide O_2_^−⋅^ and the OH^⋅^ radical,[^75^, ^76^, ^77^] whereas only the OH^⋅^ radical has been reported to play a role in the case of Fe_2_O_3_.^[78]^ Future studies can investigate this hypothesis by using different types of radical scavengers.^[79]^

These results indicate that preparing In_x_Fe_y_O_3_ solid solutions allowed achieving enhanced photocatalytic activity in the degradation of pollutants in water by tuning the band gap of the materials. Among the In_x_Fe_y_O_3_ solid solutions, In_1.7_Fe_0.3_O_3_ displayed the highest photocatalytic activity in the degradation of both probe pollutants, whereas In_1.5_Fe_0.5_O_3_ was the least active photocatalyst and the one that showed the most marked drop in activity between the test performed with UV radiation and the one with visible light. This is attributed to the incomplete formation of a solid solution in this material, with probable presence of amorphous iron oxide regions in the structure (*vide supra*), which may promote the recombination of the electron‐hole pairs formed upon irradiation.

**Figure 8:**
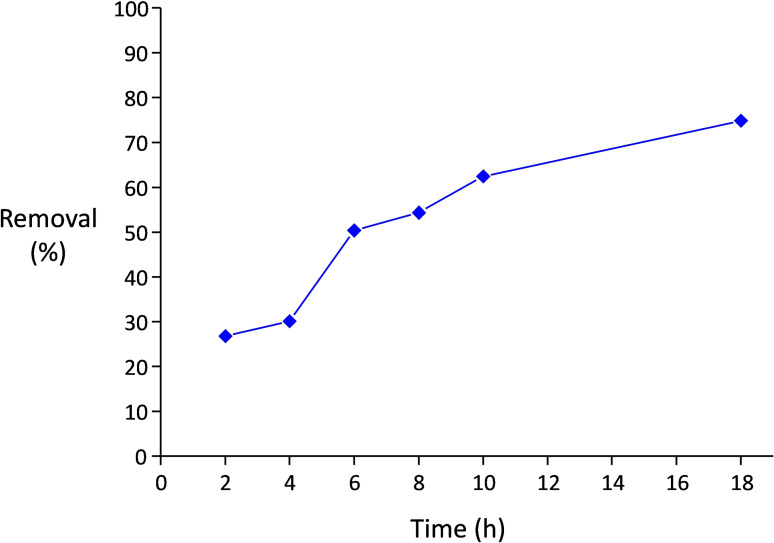
Kinetic study for the degradation of methylene blue (10 ppm, 5 ml) with 15 mg of In_1.7_Fe_0.3_O_3_ as photocatalyst, under visible light irradiation. Each point corresponds to a separate experiment. Before each period of irradiation, the mixture was stirred 1 h in the dark. The tests were performed at 35 °C in aerobic conditions.

**Figure 9:**
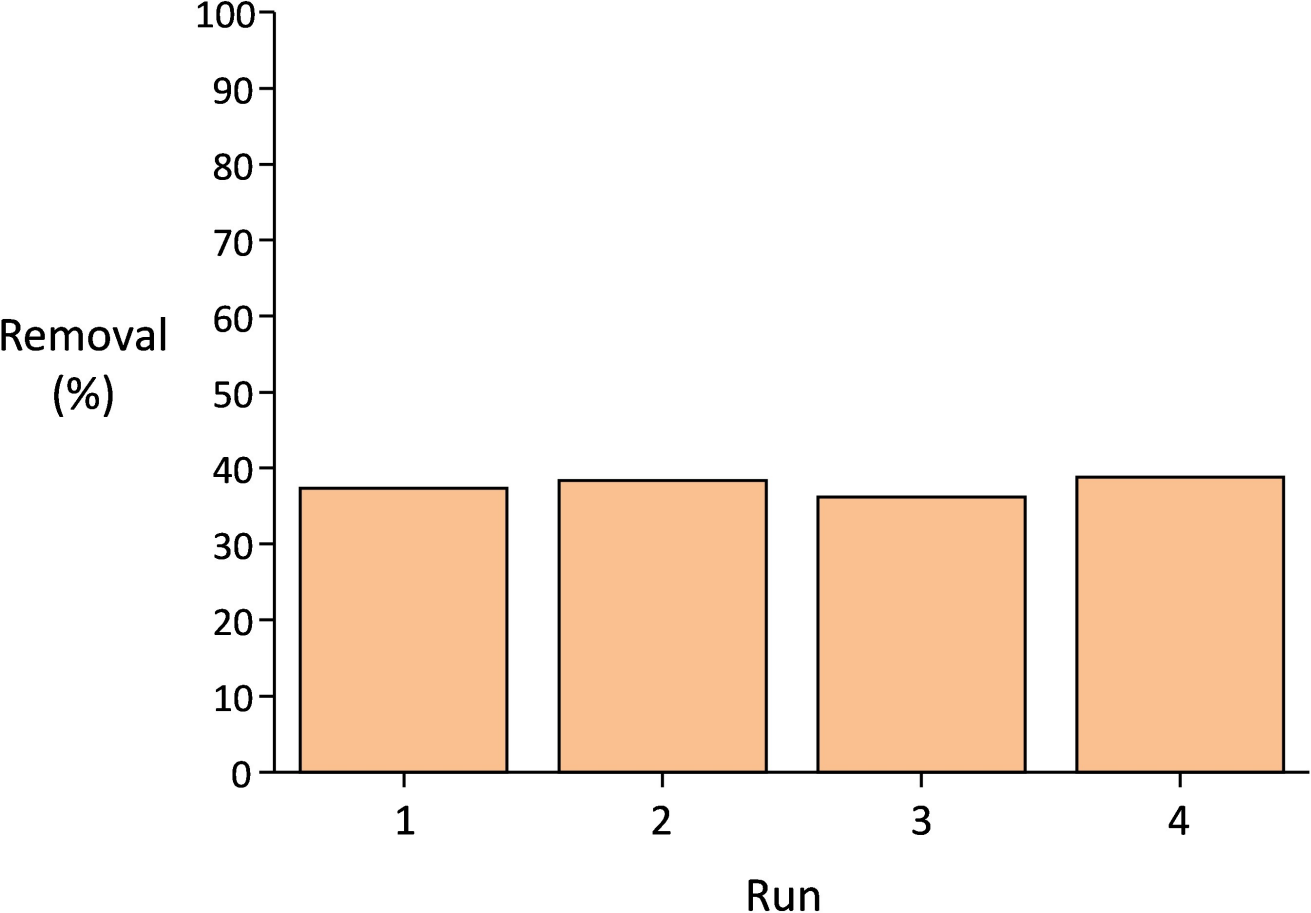
Recycling tests with 15 mg of In_1.7_Fe_0.3_O_3_ as photocatalyst, phenol (50 ppm, 5 ml) as probe pollutant, under visible light irradiation. The tests consist in 1 h of adsorption in the dark followed by 3 h of irradiation with visible light. The tests were performed at 35 °C in aerobic conditions.

Since In_1.7_Fe_0.3_O_3_ was the most active photocatalyst among the prepared In_x_Fe_y_O_3_ solid solutions, we selected it for a more detailed investigation of the photocatalytic performance through kinetic and recycling tests. A kinetic test under visible light irradiation was performed with methylene blue as probe compound. The degradation proceeded upon irradiation, achieving 75 % removal of the pollutant after 18 h (Figure 8). Since no plateau was reached in this test, it is expected that complete degradation could be attained if the irradiation is maintained for prolonged time. Importantly, In_1.7_Fe_0.3_O_3_ could be reused in four consecutive photocatalytic tests under visible light irradiation for the degradation of phenol with no loss of activity (Figure 9). Moreover, the particles could be readily recovered at the end of the reaction upon a short centrifugation step, which represents an advantage compared to TiO_2_ P25, for which centrifugation at higher speed and/or longer time is required to collect the photocatalyst particles.

## Conclusions

A series of In_2_O_3_‐Fe_2_O_3_ nanosized solid solutions in the indium‐rich region (In_x_Fe_y_O_3_, with x + y = 2; x = 1.9, 1.7, 1.5) was synthesized by a straightforward co‐precipitation method, with the purpose of engineering the band gap of the obtained materials to increase the visible light absorption compared to In_2_O_3_ and to improve the band positions compared to Fe_2_O_3_. The formation of the desired solid solutions was demonstrated by XRD, which proved the decrease in the cell parameter of the materials due to the incorporation of iron in the indium oxide framework. The formation of solid solutions led to the desired intermediate optical properties between those of the two pure oxides: the band gap energies of the In_x_Fe_y_O_3_ materials are all in the visible region, with the values gradually decreasing with increasing Fe content. The controlled co‐precipitation method allowed preparing the In_x_Fe_y_O_3_ solid solutions in the form of nanoparticles with a size of around 30–40 nm, as evidenced by TEM, which led to a maximum specific surface area of 45 m^2^/g. The combination of the tailored band gap and the nanostructuring proved beneficial for the photocatalytic performance in the degradation of pollutants under visible light, with the optimum solid solution, In_1.7_Fe_0.3_O_3_, displaying higher activity with both probe pollutants (phenol and methylene blue) compared to In_2_O_3_ and Fe_2_O_3_. The In_1.7_Fe_0.3_O_3_ photocatalyst was recycled in consecutive runs without any detectable loss of activity. In summary, we proved for the first time that preparing In_2_O_3_‐Fe_2_O_3_ solid solutions is an effective strategy to tune the band gap of these materials, leading to improved photocatalytic activity. This novel strategy is expected to be of general applicability and thus be potentially useful for the synthesis of new classes of semiconductor‐based photocatalysts with superior activity under visible light irradiation. Future work can also aim at extending the photocatalytic application of these In_x_Fe_y_O_3_ solid solutions to water splitting, biomass upgrading and fuel generation under solar radiation.[^80^, ^81^]

## Experimental Section

### Synthesis of the Solid Solutions

Indium (III) nitrate hydrate and iron (III) nitrate nonahydrate (In(NO_3_)_3_⋅xH_2_O and Fe(NO_3_)_3_⋅9H_2_O, Alfa Aesar) were used as sources of indium and iron, respectively. Ammonium carbonate ((NH_4_)_2_CO_3_, Sigma Aldrich) was used as precipitating agent. All the compounds were used without further purification. In a typical synthesis, each nitrate was dissolved in deionized water to produce an aqueous 0.1 M solution. Monometallic and mixed oxides of iron and indium were prepared mixing the solutions containing the nitrates in the desired proportions, followed by slow addition of a 1.0 M aqueous solution of ammonium carbonate. The pH variation was monitored by pH paper. The precipitation started once the pH reached a value of 8. The reaction mixture was stirred for 24 h at room temperature, then the precipitate was extensively washed with deionized water (safety note: this step is important for removing ammonium nitrate that might be present in the precipitate). The solid was dried at 80 °C overnight and finally calcined in air at 700 °C for 5 h (heating rate: 2 °C/min).^[82]^ The materials were labeled as In_x_Fe_y_O_3_ (x + y = 2; x = 0, 1.2, 1.5, 1.7, 1.9, 2).

### Characterization

The materials were characterized by a combination of techniques. Transmission electron microscopy (TEM) was performed to analyze the particle dimension and morphology. The samples were finely ground in a mortar and dispersed in ethanol. Few drops of the suspension were deposited on a 300 mesh carbon‐coated copper grid and analyzed at 200 kV on a CM‐200 FEG Philips equipment. Chemical composition was determined by energy dispersive X‐ray spectroscopy (EDX) on a scanning emission microscopy (SEM) apparatus. The SEM instrument was a Philips XL30 FEG with Genesis EDAX software. The powder samples were deposited on a carbon coated aluminum stub and covered with an Au−Pd alloy layer (60 % Au) into a Balzers sputtering device (sputtering conditions: 1 min, pressure of 0.15 torr, Ar atmosphere, 20 mA). The analysis was performed at an acceleration voltage of 10 kV. The crystal structure of the prepared materials was analyzed by X‐ray diffraction (XRD) on a STOE Stadi P instrument with Cu Kα radiation (*λ* = 1.5418 Å). Nitrogen physisorption was performed on a Micromeritics Tristar 3000 apparatus at 77 K. The specific surface area was obtained by the BET method and the pore/void size distribution was calculated from the adsorption branch of the isotherm using the BJH method.^[83]^ The optical properties of the materials were determined by solid state UV‐Vis spectrometry on a Varian Cary 5000 instrument equipped with a diffusive reflectance accessory. The band gap of the solids was determined with the Tauc plot method for a direct transition.[^40^, ^84^] The function [F(R) hν]^2^ was plotted against hν (photon energy expressed in eV) and the band gap was obtained by extrapolation of the linear part of the function on the x‐axis. The Kubelka‐Munk function F(R) was defined as (1−R)^2^/2R, where R stands for reflectance (R = 1‐ Absorbance).^[85]^

### Photocatalytic Tests

The degradation of two probe compounds in water solution was chosen as test for the photocatalytic activity. Phenol and methylene blue were dissolved in milli‐Q water (resistivity of 18.2 MΩ⋅cm) to obtain a concentration of 50 ppm (mg/L) for phenol and 10 ppm (mg/L) for methylene blue. The concentration of phenol was chosen to be higher than the one of methylene blue to facilitate its quantification by GC. 15 mg of photocatalyst were suspended in 5 ml of the aforementioned solutions before starting the test. The photocatalytic tests consisted of two steps: first the suspension was stirred for 1 h in the dark to achieve equilibrium between adsorption and desorption of the probe compound. Then, the irradiation period started, during which the photocatalytic process takes place. The tests were performed in a high‐throughput photoreactor that allows testing up to 16 samples at the same time. The photoreactor was equipped with a rotating carousel that ensures homogeneous irradiation of all the samples. The temperature in the photoreactor was set at 35 °C and the irradiation was provided by 12 lamps: Hitachi FL8BL−B were used for UV radiation (98 % UV−A and ≈2 % of visible and NIR radiation) and Sylvania cool white for visible light (90 % visible light, ≈6 % UV−A and ≈4 % NIR). Once the desired reaction time was reached, the lamps were switched off, then the photocatalyst was separated by centrifugation (3500 rpm for 5 min) and the supernatant was analyzed to quantify the residual probe compound. In the case of methylene blue, the supernatant was analyzed by UV‐Vis spectroscopy on a Perkin Elmer Lambda 12 UV‐Vis spectrophotometer and the concentration of the residual probe compound was determined by Lambert‐Beer law based on the absorption peak at 665 nm. The conversion of phenol was evaluated by gas chromatography (GC) on a Trace GC Ultra with Por. Q column and Ultra‐Fast Module (UFM)^[86]^ using tert‐butanol as internal standard, which was added after the photocatalyst was separated. For the recycling experiments, after separating the photocatalyst the solid was washed with water (3 times, 5 ml) and ethanol (3 times, 5 ml), then dried overnight at 80 °C before being reused in a photocatalytic test following the same procedure described above. The kinetic study was performed over 16 h of irradiation. Each data point of the kinetic curve corresponds to a separate experiment with the chosen time and the remaining conditions being the same as described before. Along with the newly synthesized materials, tests with iron oxide, indium oxide and the benchmark TiO_2_ P25 were performed, as well as control experiments without irradiation (i.e. 4 h in the dark). The use of TiO_2_ P25 as a benchmark is aimed at promoting reliable comparison with literature data, as this material (which consists for 75 % of anatase, 20 % of rutile and traces of amorphous titania[^15^, ^16^]) is a commercial, well‐known reference photocatalyst.^[72]^ Having a benchmark is important because the photocatalytic performance is not only determined by the intrinsic properties of the photocatalyst, but also by the test conditions (e.g. photocatalyst loading) and by the features of the photoreactor (e.g. position and number of the lamps, shape of the vials and type of stirring).

## Conflict of Interests

The authors declare no conflict of interest.

1

## Supporting information

As a service to our authors and readers, this journal provides supporting information supplied by the authors. Such materials are peer reviewed and may be re‐organized for online delivery, but are not copy‐edited or typeset. Technical support issues arising from supporting information (other than missing files) should be addressed to the authors.

Supporting Information

## Data Availability

The data that support the findings of this study are available from the corresponding author upon reasonable request.

## References

[1] A. Fujishima , K. Honda , Nature 1972, 238, 37. 12635268 10.1038/238037a0

[2] S. C. Roy , O. K. Varghese , M. Paulose , C. A. Grimes , ACS Nano 2010, 4, 1259. 20141175 10.1021/nn9015423

[3] Y. Cui , A. Labidi , X. Liang , X. Huang , J. Wang , X. Li , Q. Dong , X. Zhang , S. I. Othman , A. A. Allam , D. W. Bahnemann , C. Wang , ChemSusChem 2024, 17, e202400551. 38618906 10.1002/cssc.202400551

[4] A. Kudo , Y. Miseki , Chem. Soc. Rev. 2009, 38, 253. 19088977 10.1039/b800489g

[5] S. Malato , P. Fernandez-Ibanez , M. I. Maldonado , J. Blanco , W. Gernjak , Catal. Today 2009, 147, 1.

[6] S. Dong , J. Feng , M. Fan , Y. Pi , L. Hu , X. Han , M. Liu , J. Sun , J. Sun , RSC Adv. 2015, 5, 14610.

[7] D. S. Bhatkhande , V. G. Pangarkar , A. Beenackers , J. Chem. Technol. Biotechnol. 2011, 77, 102.

[8] E. Pelizzetti , C. Minero , Electrochim. Acta 1993, 38, 47.

[9] S. Yang , L. Lou , K. Wang , Y. Chen , Appl. Catal. A: General 2006, 301, 152.

[10] M. R. Hoffmann , S. T. Martin , W. Choi , D. W. Bahnemann , Chem. Rev. 1995, 95, 69.

[11] J. Tian , Z. Zhao , A. Kumar , R. I. Boughton , H. Liu , Chem. Soc. Rev. 2014, 43, 6920. 25014328 10.1039/c4cs00180j

[12] Y. Lan , Y. Lu , Z. Ren , Nano Energy 2013, 2, 1031.

[13] C. Aprile , A. Corma , H. Garcia , Phys. Chem. Chem. Phys. 2008, 10 (6), 769. 18231679 10.1039/b712168g

[14] Q. Guo , C. Zhou , Z. Ma , X. Yang , Adv. Mater. 2019, 31, 1901997. 10.1002/adma.201901997 31423680

[15] M. E. Simonsen , H. Jensen , Z. Li , E. G. Sogaard , J. Photochem. Photobiol. A 2000, 200, 192.

[16] R. I. Bickley , T. Gonzales-Carreno , J. S. Lees , L. Palmisano , R. J. D. Tilley , J. Solid State Chem. 1991, 92, 178.

[17] M. Ismael , A. Sharma , N. Kumar , Sust. Mater. Tech. 2024, 40, e00826.

[18] M. Pelaez , N. T. Nolan , S. C. Pillai , M. K. Seery , P. Falaras , A. G. Kontos , P. S. M. Dunlop , W. J. Hamilton , J. A. Byrne , K. O′Shea , M. H. Entezari , D. D. Dionysiou , Appl. Catal. B 2012, 125, 331.

[19] R. Asahi , T. Morikawa , T. Ohwaki , K. Aoki , Y. Taga , Science 2011, 293, 269. 10.1126/science.1061051 11452117

[20] J. Choi , H. Park , M. R. Hoffmann , J. Phys. Chem. C 2010, 114, 783.

[21] D. Cani , J. C. van der Waal , P. P. Pescarmona , Appl. Catal. A 2021, 621, 118179.

[22] M. Ismael , Sol. Energy 2020, 211, 522.

[23] A. Primo , A. Corma , H. Garcia , Phys. Chem. Chem. Phys. 2011, 13, 886. 21085723 10.1039/c0cp00917b

[24] H. G. Kim , D. W. Hwang , J. S. Lee , J. Am. Chem. Soc. 2004, 126, 8912. 15264819 10.1021/ja049676a

[25] Z. Zou , J. Ye , K. Sayama , H. Arakawa , Nature 2001, 414, 625. 11740556 10.1038/414625a

[26] A. Kudo , Y. Miseki , Chem. Soc. Rev. 2009, 8, 253. 10.1039/b800489g 19088977

[27] P. Xu , G. M. Zeng , D. L. Huang , C. L. Feng , S. Hu , M. H. Zhao , C. Lai , Z. Wei , C. Huang , G. X. Xie , Z. F. Liu , Sci. Total Environ. 2012, 424, 1. 22391097 10.1016/j.scitotenv.2012.02.023

[28] M. Mishra , D. M. Chun , Appl. Catal. A 2015, 498, 126–141.

[29] P. Zhang , T. Wang , J. Gong , Chem. 2018, 4, 223.

[30] C. N. C. Hitam , A. A. Jalil , J. Environ. Manage. 2020, 258, 110050. 31929077 10.1016/j.jenvman.2019.110050

[31] T. Ohmori , H. Takahashi , H. Mametsuka , E. Suzuki , Phys. Chem. Chem. Phys. 2000, 2, 3519.

[32] K. Sivula , F. Le Formal , M. Grätzel , ChemSusChem 2011, 4, 432. 21416621 10.1002/cssc.201000416

[33] N. J. Cherepy , D. B. Liston , J. A. Lovejoy , H. Deng , J. Z. Zhang , J. Phys. Chem. B 1998, 102, 770.

[34] A. G. Joly , J. R. Williams , S. A. Chambers , G. Xiong , W. P. Hess , J. Appl. Phys. 2006, 99, 053521.

[35] M. Bledowski , L. Wang , A. Ramakrishnan , O. V. Khavryuchenko , V. D. Khavryuchenko , P. C. Ricci , J. Strunk , T. Cremer , C. Kolbeck , R. Beranek , Phys. Chem. Chem. Phys. 2011, 13, 21511. 22057224 10.1039/c1cp22861g

[36] Z. Xu , I. Tabata , K. Hirogaki , K. Hisada , T. Wang , S. Wang , T. Hori , Mater. Lett. 2011, 65, 1252.

[37] F. E. Osterloh , Chem. Soc. Rev. 2013, 42, 2294. 23072874 10.1039/c2cs35266d

[38] M. R. Dhananjeyan , J. Kiwi , K. R. Thampi , Chem. Commun. 2000, 1443.

[39] P. Erhart , A. Klein , R. G. Egdell , K. Albe , Phys. Rev. B 2007, 75, 153205.

[40] P. D. C. King , T. D. Veal , F. Fuchs , C. Y. Wang , D. J. Payne , A. Bourlange , H. Zhang , G. R. Bell , V. Cimalla , O. Ambacher , R. G. Egdell , F. Bechstedt , C. F. Mc Conville , Phys. Rev. B 2009, 79, 205211.

[41] A. Walsh , J. L. F. da Silva , S. H. Wei , C. Körber , A. Klein , L. F. J. Piper , A. De Masi , K. E. Smith , G. Panaccione , P. Torelli , D. J. Payne , A. Bourlange , R. G. Egdell , Phys. Rev. Lett. 2008, 100, 167402. 18518246 10.1103/PhysRevLett.100.167402

[42] S. K. Poznyak , A. N. Golubev , A. I. Kulak , Surf. Sci. 2000, 454–456, 396.

[43] S. Q. Guo , X. Zhang , Z. W. Hao , G. D. Gao , G. Li , L. Liu , RSC Adv. 2014, 4, 31353.

[44] Z. Li , P. Zhang , T. Shao , J. Wang , X. Li , J. Hazard. Mater. 2013, 260, 40. 23742955 10.1016/j.jhazmat.2013.04.042

[45] B. Josh , H. Yoon , H. Kim , M. W. Kim , M. G. Mali , S. S. Al-Deyab , S. S. Yoon , RSC Adv. 2015, 5, 85323.

[46] L. Y. Chen , W. D. Zheng , Appl. Surf. Sci. 2014, 301, 428.

[47] V. Shanmuganathan , J. S. Kumar , R. Pachaiappan , P. Thangadurai , Nanoscale Adv. 2021, 3, 471. 36131727 10.1039/d0na00694g PMC9418826

[48] M. A. Khan , A. U. Khan , K. Tahir , M. S. Othman Alhar , M. E. A. Zaki , T. M. Althagafi , A. A. Alanazi , S. I. Al-Saeedi , H. S. Al-Shehri , S. Nazir , Mater. Chem. Phys. 2023, 302, 127746.

[49] M. Xu , J. Zai , Y. Yuan , X. Qian , J. Mater. Chem. 2012, 22, 23929.

[50] S. M. Ji , S. H. Choi , J. S. Jang , E. S. Kim , J. S. Lee , J. Phys. Chem. C 2009, 113, 17824.

[51] H. Liu , J. Yuan , Z. Jiang , W. Shangguan , H. Einaga , Y. Teraoka , J. Mater. Chem. 2011, 21, 16535.

[52] C. Zhang , Y. Zhu , Chem. Mater. 2005, 17, 3537.

[53] I. E. Castelli , T. Olsen , S. Datta , D. D. Landis , S. Dahl , K. S. Thygesen , K. W. Jacobsen , Energy Environ. Sci. 2012, 5, 5814.

[54] S. Anandan , N. Ohashi , M. Miyauchi , App. Catal. B: Environ. 2010, 100, 502.

[55] F. E. Oropeza , B. Davies , R. G. Palgrave , R. G. Egdell , Phys. Chem. Chem. Phys. 2011, 13, 7882. 21445426 10.1039/c0cp02639e

[56] F. Fresno , D. Tudela , J. M. Coronado , J. Soria , Catal. Today 2009, 143, 230.

[57] R. D. Shannon , Acta Crystallogr. 1976, A32, 751.

[58] S. Qaseem , S. Rizwan Ali , M. Naeem , S. Rizvi , Appl. Surf. Sci. 2015, 331, 87.

[59] M. Alagiri , S. B. Abdul Hamid , Mater. Lett. 2014, 136, 329.

[60] C. Fahed , S. B. Qadri , H. Kim , A. Pique’ , M. Miller , N. A. Mahadik , M. V. Rao , M. Osofsky , Phys. Status Solidi C 2010, 7, 2298.

[61] D. Berardan , E. Guilmeau , J. Phys. Condens. Matter 2007, 19, 236224.

[62] W. Yao , J. Ye , J. Phys. Chem. B 2006, 110, 11188. 16771382 10.1021/jp0608729

[63] M. Sorescu , L. Diamandescu , D. Tarabasanu-Mihaila , J. Phys. Chem. Solids 2004, 65, 1719.

[64] S. Z. Karazhanov , P. Ravindran , P. Vajeeston , A. Ulyashin , T. G. Finstad , H. Fjellvag , Phys. Rev. B 2007, 76, 75129.

[65] O. D. Slagle , H. A. McKinstry , Acta Crystallogr. 1966, 21, 1013.

[66] F. R. Sensato , R. Custodio , E. Longo , A. Beltran , J. Andres , Catal. Today 2003, 85, 145.

[67] K. S. W. Sing , D. H. Everett , R. A. W. Haul , L. Moscou , R. A. Pierotti , J. Rouquerol , T. Siemieniewska , Pure Appl. Chem. 1985, 57, 603.

[68] F. Amano , K. Nogami , M. Tanaka , B. Ohtani , Langmuir 2010, 26, 7174. 20141124 10.1021/la904274c

[69] G. Riegel , J. R. Bolton , J. Phys. Chem. 1995, 99, 4215.

[70] S. Venkataprasad Bhat , F. L. Deepak , Solid State Commun. 2005, 135, 345.

[71] S. Bae , S. Kim , S. Lee , W. Choi , Catal. Today 2014, 224, 21.

[72] D. Cani , P. P. Pescarmona , J. Catal. 2014, 311, 404.

[73] X. Yan , K. Nishijima , R. Abe , B. Ohtani , Chem. Phys. Lett. 2006, 429, 606.

[74] D. M. S. Marcolongo , F. Nocito , N. Ditaranto , R. Comparelli , M. Aresta , A. Dibenedetto , Catalysts 2022, 12, 153.

[75] R. Xie , K. Fang , Y. Liu , W. Chen , J. Fan , X. Wang , Y. Ren , Y. Song , J. Mater. Sci. 2020, 55, 11919.

[76] Y. Xing , W. Que , X. Yin , Z. He , X. Liu , Y. Yang , J. Shao , L. B. Kong , Appl. Surf. Sci. 2016, 387, 36.

[77] S. Tang , X. Zhang , S. Li , C. Zheng , H. Li , X. Xiao , ACS Omega 2023, 8, 40099. 37929117 10.1021/acsomega.3c02652 PMC10620787

[78] A. Trenczek-Zajac , M. Synowiec , K. Zakrzewska , K. Zazakowny , K. Kowalski , A. Dziedzic , M. Radecka , ACS Appl. Mater. Interfaces 2022, 14, 38255. 35969717 10.1021/acsami.2c06404 PMC9412959

[79] T. Giousis , S. Fang , M. Miola , S. Li , A. Lazanas , M. Prodromidis , E. K. Tekelenburg , D. Moschovas , M. A. Loi , P. Rudolf , D. Gournis , P. P. Pescarmona , J. Environ. Chem. Eng. 2023, 11, 109784.

[80] W. Zhang , X. Li , S. Liu , J. Qiu , J. An , J. Yao , S. Zuo , B. Zhang , H. Xia , C. Li , ChemSusChem 2022, 15, e202102158. 34914202 10.1002/cssc.202102158

[81] X. Du , Y. Peng , J. Albero , D. Li , C. Hu , H. García , ChemSusChem 2022, 15, e202102107. 34841693 10.1002/cssc.202102107

[82] M. I. Ivanovskaya , D. A. Kotsikau , A. Taurino , P. Siciliano , Sensor Actuat. B 2007, 124, 133.

[83] S. Brunauer , P. H. Emmett , E. Teller , J. Am. Chem. Soc. 1938, 60, 309.

[84] T. Abe , Y. Tachibana , T. Uematsu , M. Iwamoto , Chem. Commun. 1995, 1617.

[85] J. Tauc , R. Grigorovici , A. Vancu , Phys. Status Solidi 1966, 15, 627.

[86] Li Li , D. Cani , P. P. Pescarmona , Inorg. Chim. Acta 2015, 431, 289.

