# Supplementary material for: Indium‐Iron Oxide Nanosized Solid Solutions as Photocatalysts for the Degradation of Pollutants under Visible Radiation
Source: ChemSusChem. 2024 Nov 7;18(3):e202401180. doi: 10.1002/cssc.202401180 (PMC11790003; doi:10.1002/cssc.202401180)
Supplement: Supplementary file 1 — Supporting Information [file CSSC-18-e202401180-s001.pdf]

ChemSusChem

Supporting Information

# Indium-Iron Oxide Nanosized Solid Solutions as Photocatalysts for the Degradation of Pollutants under Visible Radiation

Damiano Cani, Timo Cuyvers, and Paolo P. Pescarmona*

# Indium-iron oxide nanosized solid solutions as photocatalysts for the degradation of pollutants under visible radiation

Damiano Cani[a], Timo Cuyvers[a], Paolo P. Pescarmona[b,*]

[a] Centre for Surface Chemistry and Catalysis, University of Leuven (KU Leuven), Kasteelpark Arenberg 23, 3001 Heverlee, Belgium.

[b] Chemical Engineering Group, Engineering and Technology Institute Groningen (ENTEG), University of Groningen, Nijenborgh 4, 9747 AG, Groningen, The Netherlands.

[*] Corresponding author: p.p.pescarmona@rug.nl

## Supporting information

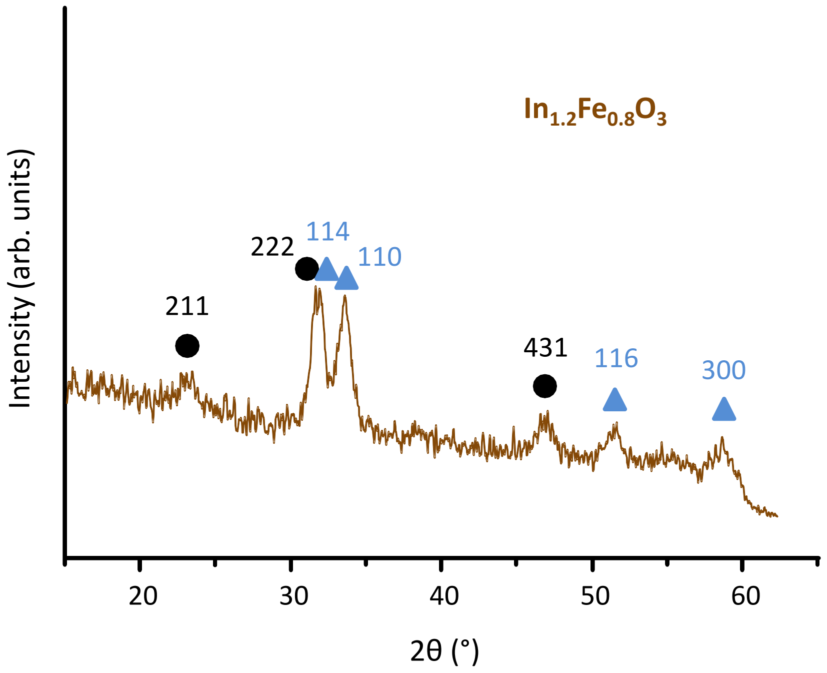


**Fig. S1.** Diffractogram of $In_{1.2}Fe_{0.8}O_3$ (60 mol% $In_2O_3$ and 40 mol% $Fe_2O_3$) showing the presence of the peaks of α-$Fe_2O_3$ (▲) and $In_2O_3$ (●). $In_2O_3$: file 00-006-0416 PDF-2008. α-$Fe_2O_3$: file 01-088-2359 PDF-2008.

## Lattice parameters of solid solutions

Several equations have been proposed for evaluating the variation of lattice parameters in solid solutions as a function of the molar fraction and the lattice parameters of the components. The general correlation between the lattice parameters of the components and the lattice parameter of the solid solution is given by $a_{12}^n = a_1^n c_1 + a_2^n c_2$ [1], where $a_{12}$, $a_1$ and $a_2$ are the lattice parameters of the solid solution and of the two pure oxides, respectively, and $c_1$, $c_2$ are the molar fractions of the two oxides. The exponent $n$ is an arbitrary integer that depends on the specific equation used to describe the system. Vegard's law predicts a linear relationship between the lattice parameter and the concentration of the components, thus $n$ = 1. This is the simplest correlation and most of the solid solutions have been found to deviate from this ideal behavior [2]. Alternative values have been proposed based on different considerations, such as $n$ = 3 (Retger's law), or $n$ = 8 (Grimm's law) [1].

The lattice parameters are determined from the $d$-spacing obtained by XRD analysis. For a cubic crystal system, the relation between the lattice parameter and the $d$-spacing is:

$$\frac{1}{d^2} = \frac{h^2 + k^2 + l^2}{a^2}$$

The value of the lattice parameter is thus given by:

$$a = \sqrt{(h^2 + k^2 + l^2)d^2}$$

where *h*, *k*, *l* correspond to the indices of the peak under investigation, *d* is the *d*-spacing and *a* is the lattice parameter.

The theoretical values of $a_{In_2O_3}$ and $a_{Fe_2O_3}$ for the cubic crystal system are 10.08573Å (file 00-006-0416 PDF-2008) and 9.404 Å (file 00-039-0238 PDF-2008), respectively.

The theoretical correlation between the lattice parameters and the composition of the material is obtained by 3 different approaches following the laws of Vegard, Retger and Grimm.

The general equation used to evaluate the theoretical value of the lattice parameter is:

$$a_{In_xFe_yO_3} = \sqrt[n]{\left[a_{In_2O_3}{}^n \cdot c_{In_2O_3}\right] + \left[a_{Fe_2O_3}{}^n \cdot c_{Fe_2O_3}\right]}$$

where $a_{In_xFe_yO_3}$ is the lattice parameter of the solid solution; $a_{In_2O_3}$ and $a_{Fe_2O_3}$ the lattice parameters of pure indium and iron oxide, respectively; $c_{In_2O_3}$ and $c_{Fe_2O_3}$ the molar fractions of indium and iron oxide in the solid solution; *n* represents a parameter that takes the value of 1 (Vegard's law), 3 (Retger's law) or 8 (Grimm's law).

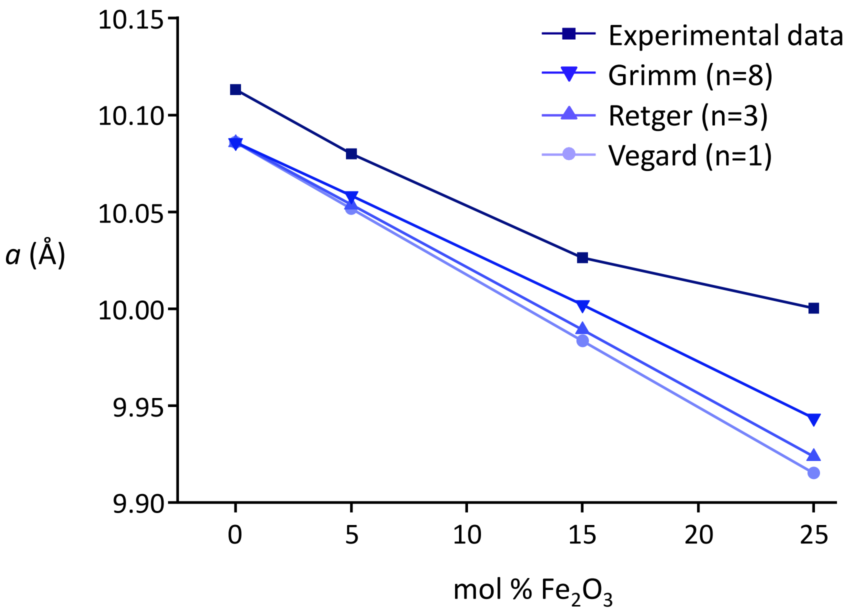


**Fig. S2.** Lattice parameter *a* (Å) evaluated from the (400) peak in the $In_xFe_yO_3$ series (with y = 0, 0.1, 0.3, 0.5). The experimental values are plotted together with the expected values based on three different theoretical equations (Vegard, Retger, Grimm).

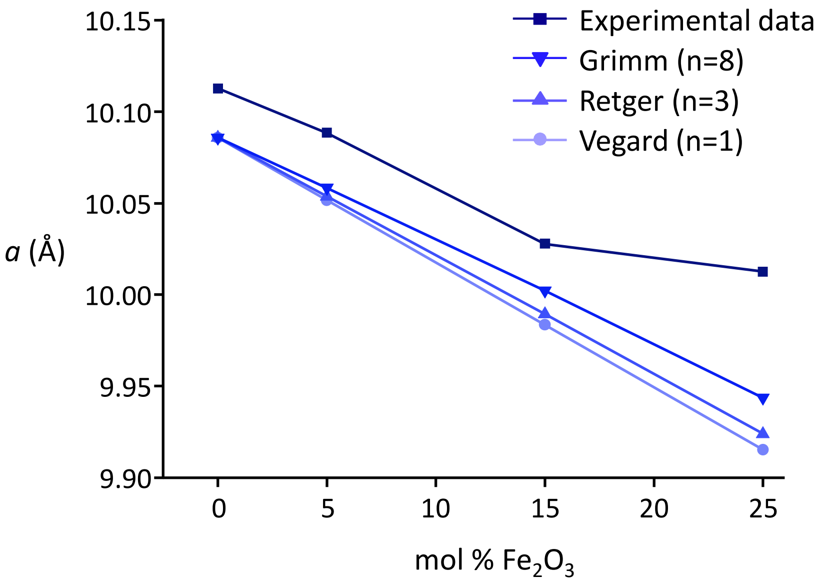


**Fig. S3.** Lattice parameter *a* (Å) evaluated from the (440) peak in the $In_xFe_yO_3$ series (with y = 0, 0.1, 0.3, 0.5). The experimental values are plotted together with the expected values based on three different theoretical equations (Vegard, Retger, Grimm).

## References


[1] O.D. Slagle, H.A. McKinstry, Acta Cryst, 21 (1966) 1013.

[2] F.R. Sensato, R. Custodio, E. Longo, A. Beltran, J. Andres, Catal. Today, 85 (2003) 145.
